# Transcriptome analysis provides new insights into the transcriptional regulation of methyl jasmonate-induced flavonoid biosynthesis in pear calli

**DOI:** 10.1186/s12870-020-02606-x

**Published:** 2020-08-25

**Authors:** Apekshika T. Premathilake, Junbei Ni, Jiaqi Shen, Songling Bai, Yuanwen Teng

**Affiliations:** 1 College of Agriculture and Biotechnology, Zhejiang University, Hangzhou, 310058 Zhejiang Province China; 2 The Key Laboratory of Horticultural Plant Growth, Development and Quality Improvement, the Ministry of Agriculture of China, Hangzhou, 310058 Zhejiang Province China; 3 Zhejiang Provincial Key Laboratory of Integrative Biology of Horticultural Plants, Hangzhou, 310058 Zhejiang Province China; 4 Department of Export Agriculture, Uva Wellassa University, Badulla, 90000 Sri Lanka

**Keywords:** Methyl jasmonate, Flavonoid, Transcriptome, WGCNA, PcMYB10, PcMYC2

## Abstract

**Background:**

Flavonoid biosynthesis is strongly influenced by phytohormones. For example, methyl jasmonate (MeJA) enhances the flavonoid accumulation in pear. However, the molecular mechanism underlying the MeJA-induced flavonoid biosynthesis in pear is largely uncharacterized. Therefore, the transcriptome of pear calli treated with MeJA was analyzed to elucidate the mechanism regulating MeJA-mediated flavonoid biosynthesis.

**Results:**

The application of exogenous MeJA significantly enhanced flavonoid accumulation, especially anthocyanin, in pear calli. A weighted gene co-expression network analysis identified the differentially expressed genes associated with MeJA-induced flavonoid biosynthesis. The MeJA treatment upregulated the expression of the flavonoid biosynthesis pathway structural genes (*PcCHS*, *PcCHI*, *PcF3H*, *PcDFR*, *PcANS*, *PcANR2a*, and *PcLAR1*). The MYB family members were the main transcription factors regulating the MeJA-induced flavonoid biosynthesis, but the bHLH, AP2-EREBP, NAC, WRKY, and TIFY families were also involved. In addition to PcMYB10, which is a known positive regulator of anthocyanin biosynthesis in pear, several novel MYB candidates that may regulate flavonol and proanthocyanidin biosynthesis were revealed. Yeast two-hybrid and bimolecular fluorescence complementation assays demonstrated that PcMYB10 and PcMYC2 can directly interact with each other and bind to JAZ repressors (PcJAZ1 and PcJAZ2).

**Conclusions:**

The PcMYB10–PcMYC2 molecular complex is likely involved in the regulation of jasmonate-mediated flavonoid biosynthesis at the transcript level. The data generated in this study may clarify the transcriptional regulatory network associated with the MeJA-induced flavonoid accumulation in pear calli and provide a solid foundation for future studies.

## Background

Flavonoids are a group of secondary metabolites that are extensively distributed in plants. They have been divided into several major subgroups such as anthocyanins, proanthocyanidins, flavonols, flavones, and isoflavones [1]. These metabolites play important biological roles specifically related to plant development and defense. Anthocyanins are water soluble pigments that are mainly involved in flower and fruit coloration. Therefore, anthocyanins are important for attracting pollinators and they also influence seed dispersal [2]. Additionally, anthocyanins are natural antioxidants [3]. Proanthocyanidins are condensed tannins and are primarily concentrated in seeds, but they also affect fruit flavor [4]. Flavonols, flavones, flavanones, and isoflavones help protect plants from ultraviolet radiation and pathogens [5]. Furthermore, flavonoids are essential for plant adaptations to biotic and abiotic stresses [6].

The flavonoid biosynthesis pathway is a branch of the phenylpropanoid pathway [7] and requires several enzymes. For example, genes encoding PAL (phenylalanine ammonia lyase), CHS (chalcone synthase), CHI (chalcone isomerase), and F3H (flavanone 3-hydroxylase) are the early biosynthetic genes (EBGs) that produce common precursors in the early steps of the pathway [8]. The late biosynthetic genes (LBGs) contribute to a later stage, during which specific flavonoid products are synthesized such as anthocyanins, proanthocyanidins, and flavonols. The LBGs include those encoding DFR (dihydroflavonol 4-reductase), ANS (anthocyanin synthase), and UFGT (UDP-glucose:flavonoid 3-glucosyltransferase), which are specifically involved in anthocyanin biosynthesis [9]. In contrast, LAR (leucoanthocyanidin reductase) and ANR (anthocyanin reductase) are key enzymes mediating proanthocyanidin biosynthesis [10]. Additionally, FLS (flavonol synthase) is specific for flavonol biosynthesis [11]. The structural genes of the flavonoid biosynthesis pathway are transcriptionally controlled by the MYB–bHLH–WDR (MBW) complex comprising a MYB transcription factor, a basic helix-loop-helix (bHLH), and a WD-repeat protein [12].

Flavonoid biosynthesis is affected by various factors, including light [13], temperature [14], water deficit [15], and nutrient deficiency [16]. Moreover, phytohormones are among the most important regulators of the biosynthesis of flavonoid compounds in plants. The effects of plant hormones, such as jasmonate [17, 18], abscisic acid [19, 20], auxin [21], ethylene [22], cytokinin [23], and gibberellin [24], on flavonoid accumulation have been widely studied.

Jasmonates are oxylipins (oxygenated fatty acids) synthesized by the octadecanoid/hexadecanoid pathways [25]. Jasmonic acid can be metabolized to several derivatives, including methyl jasmonate (MeJA), jasmonoyl-isoleucine (JA-Ile), jasmonyl-1-aminocyclopropane-1-carboxylic acid (JA-ACC), glucosylated derivatives of JA (e.g., JA-O-Glc), and cis-jasmone. However, of these derivatives, only MeJA and JA-Ile have been well characterized [26]. Multiple studies have revealed that MeJA application induces flavonoid biosynthesis in different fruit species such as apple (*Malus domestica*) [27], grape [28], blueberry [29], and strawberry (*Fragaria × ananassa*) [30]. In pear, the post-harvest application of MeJA induces anthocyanin accumulation in the fruit peel under UV-B/Vis irradiation [31]. In addition to anthocyanin, Ni et al. [22] reported that MeJA increases the accumulation of other flavonoid derivatives, including flavone and isoflavone, in pear fruit.

The molecular mechanism underlying jasmonate-induced anthocyanin accumulation has been clarified in *Arabidopsis thaliana* (Arabidopsis) and apple [17, 32, 33]. Jasmonate ZIM-domain proteins (JAZs) are substrates of the SCF^COI1^ complex and negatively regulate the jasmonate signaling pathway [34, 35]. The JAZ proteins can directly interact with MYB and bHLH and disrupt the formation of the MBW complex [32, 36]. After the jasmonate signal is perceived, JAZ proteins are recruited by COI1 to the SCF^COI1^ complex for ubiquitination and are subsequently degraded by the 26S proteasome pathway [32]. This triggers the release of MYB and bHLH transcription factors and the formation of the MBW complex to activate the expression of flavonoid biosynthesis pathway structural genes [18, 33]. The expression levels of MYB and bHLH transcription factor genes are upregulated by MeJA in Arabidopsis and apple, suggesting these transcription factors are regulated by the jasmonate signaling pathway. However, the molecular mechanism associated with MeJA-induced flavonoid biosynthesis in pear is largely unknown. Therefore, in the present study, pear calli treated with MeJA underwent a comprehensive transcriptome analysis to identify the differentially expressed genes (DEGs) between the MeJA-treated and untreated control pear calli. Moreover, a co-expression network was constructed to detect the transcripts specifically related to MeJA-induced flavonoid biosynthesis. This study generated a pool of candidate genes that should be analyzed in greater detail to clarify the molecular mechanism associated with MeJA-induced flavonoid biosynthesis in pear. Specifically, we examined pear calli because of their lack of seasonal restrictions and the ease in which their gene effects can be observed in a homogeneous system, which can substantially accelerate the study of gene functions in pear.

## Results

### MeJA-induced flavonoid accumulation in pear calli

**Fig. 1:**
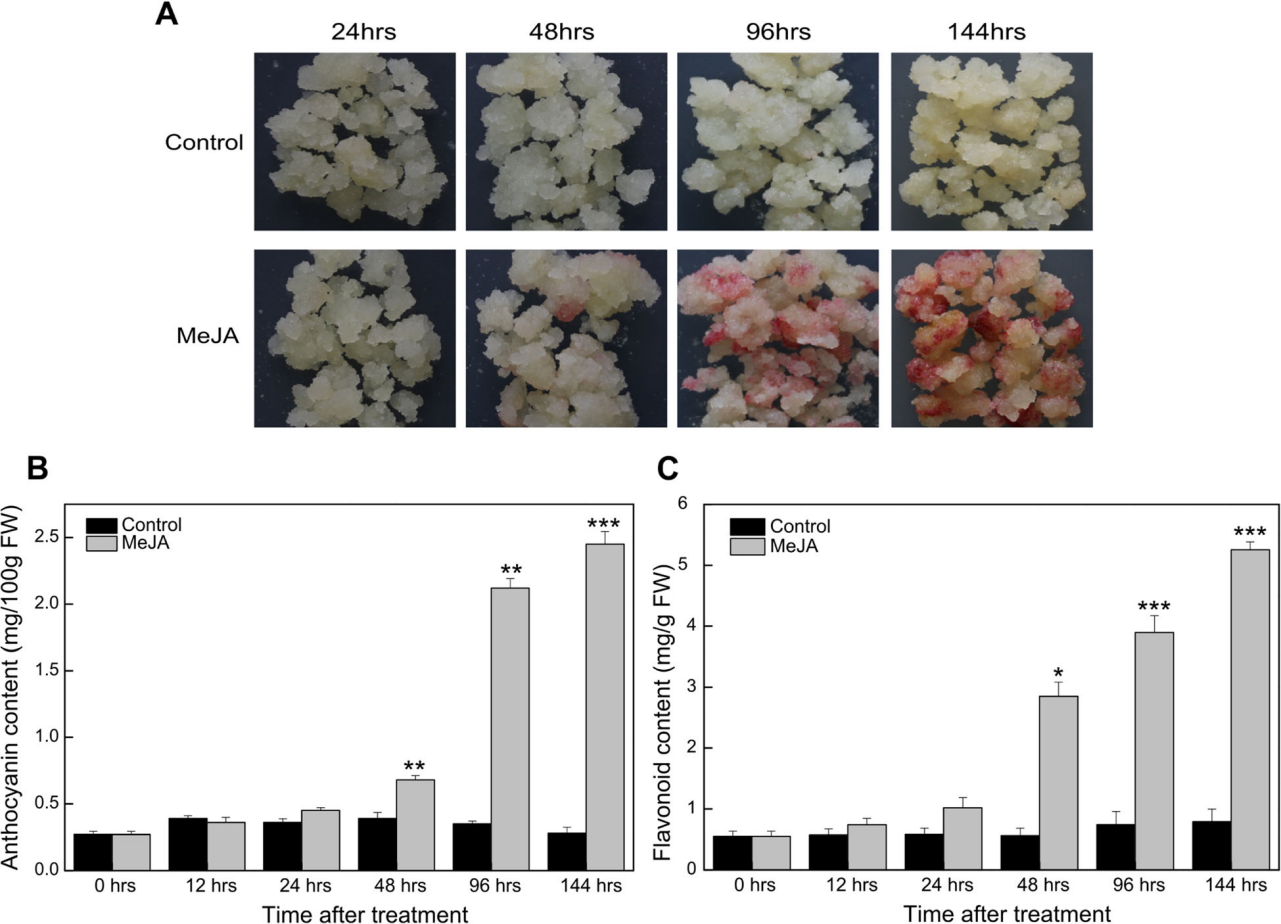
Effects of a MeJA treatment on anthocyanin and flavonoid accumulation in pear calli. **a** Phenotypic comparison of the pear calli treated with MeJA (50 μmol/L) and the control calli (1% methanol). **b** Anthocyanin contents of the MeJA-treated and control pear calli. **c** Flavonoid contents of the MeJA-treated and control pear calli

To assess the effect of MeJA on flavonoid biosynthesis, pear calli were transferred to Murashige and Skoog (MS) medium containing 50 μmol/L MeJA, whereas control calli were transferred to MS medium with 1% methanol. Distinct phenotypic differences between the MeJA-treated and control pear calli were observed at 48 h after the treatment (Fig. 1a). Additionally, red coloration was detected in the MeJA-treated pear calli. The anthocyanin and flavonoid contents of the MeJA-treated pear calli increased substantially after 48 h and continued to increase for the duration of the treatment period (Fig. 1b and c).

### Overview of RNA sequencing

Total RNA was extracted from pear calli sampled at 0, 12, and 48 h after the MeJA treatment and from the corresponding control samples for an RNA sequencing (RNA-seq) analysis. The number of raw reads for each library ranged from 44.39 to 62.10 million. After the quality filtering process, 43.43 to 60.77 million clean reads were generated for each library. Additionally, the Q20 and Q30 values for all libraries were ≥ 96.48% and ≥ 90.9%, respectively, confirming the high quality of the RNA and sequencing data that were used for further analyses of gene expression. The total reference genome mapping rate varied from 70 to 72.67%, with 63.9 to 65.79% of the reads uniquely mapped (Additional file 4: Table S1).

### Analysis of differentially expressed genes

To determine the differences in gene expression between MeJA-treated and control pear calli, gene expression levels were normalized based on the fragments per kilobase per million (FPKM) values (Additional file 5: Table S2). All uniquely mapped reads were used to calculate the gene FPKM values. The DEGs were identified and filtered according to the following criteria: adjusted *p*-value < 0.005 and log_2_ (fold-change) value > 1. At 12 h after the treatment, the expression levels of 4228 and 3410 genes were upregulated and downregulated, respectively, in the MeJA-treated pear calli relative to the corresponding control levels. Furthermore, 2583 and 1659 gene expression levels were upregulated and downregulated, respectively, in the MeJA-treated pear calli compared with the control levels at 48 h after the treatment (Additional file 1: Fig. S1).

### GO annotation and KEGG pathway analyses

**Fig. 2:**
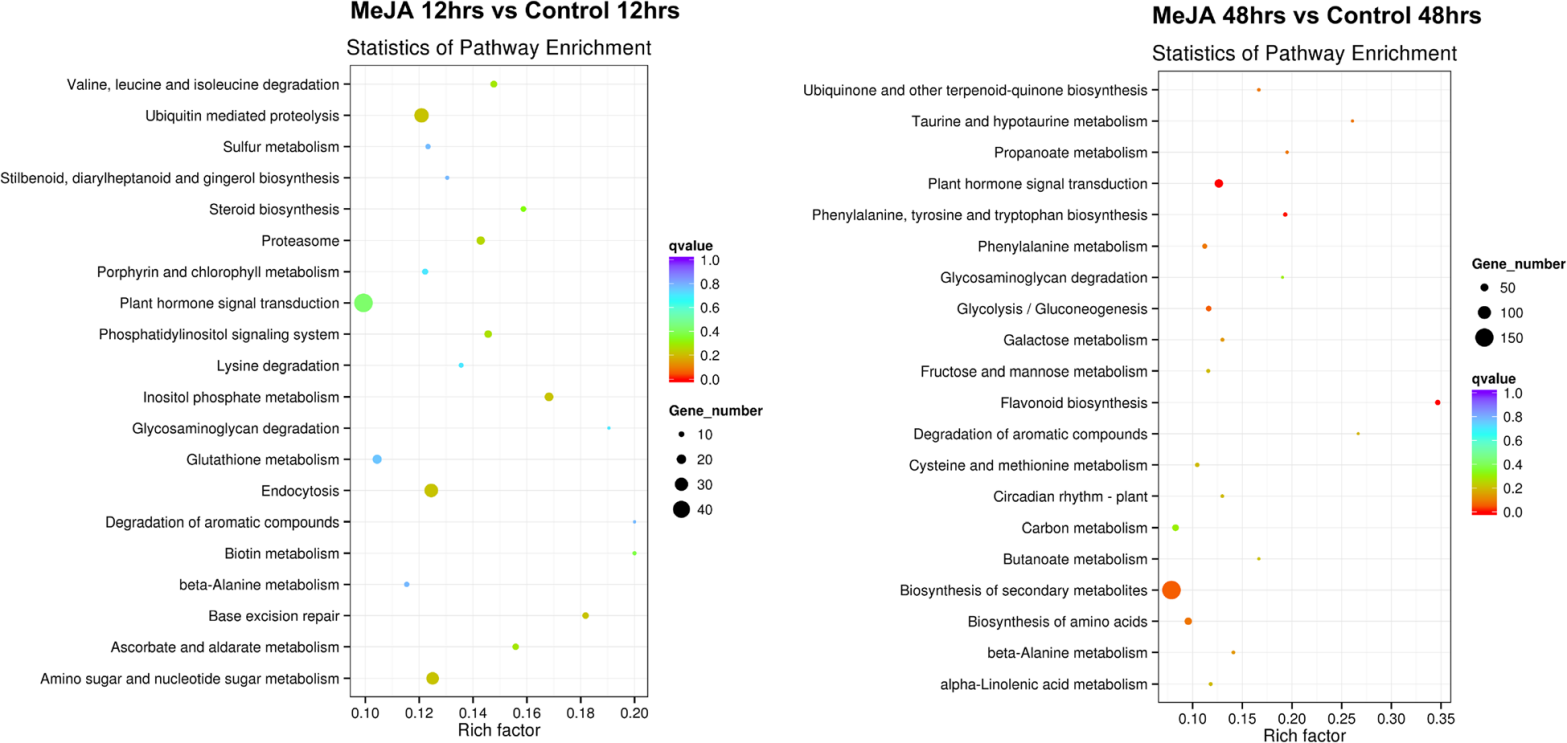
Enriched KEGG pathways among the DEGs more highly expressed in MeJA-treated pear calli than in control calli (1% methanol) after 12 and 48 h. The y-axis and x-axis present the KEGG pathways and the Rich factors, respectively. Dot size corresponds to the number of distinct genes, whereas dot color reflects the q-value

All unigenes were functionally annotated based on the Gene Ontology (GO) database. The predicted genes were grouped into the three main categories (biological process, molecular function, and cellular component). (Additional file 2: Fig. S2). Additionally, the enriched Kyoto Encyclopedia of Genes and Genomes (KEGG) pathways among the unigenes were determined to elucidate the biological pathways activated by the MeJA treatment. The functional analysis revealed that the flavonoid biosynthesis (mdm0094) pathway was significantly enhanced in the MeJA-treated pear calli at 12 and 48 h after the treatment. In addition to flavonoid biosynthesis, plant hormone signal transduction (mdm04075), biosynthesis of secondary metabolites (mdm01110), and phenylalanine, tyrosine, and tryptophan biosynthesis (mdm00400) were also significantly enhanced in the MeJA-treated pear calli compared with the control (Fig. 2).

### Jasmonate signal transduction pathway

**Fig. 3:**
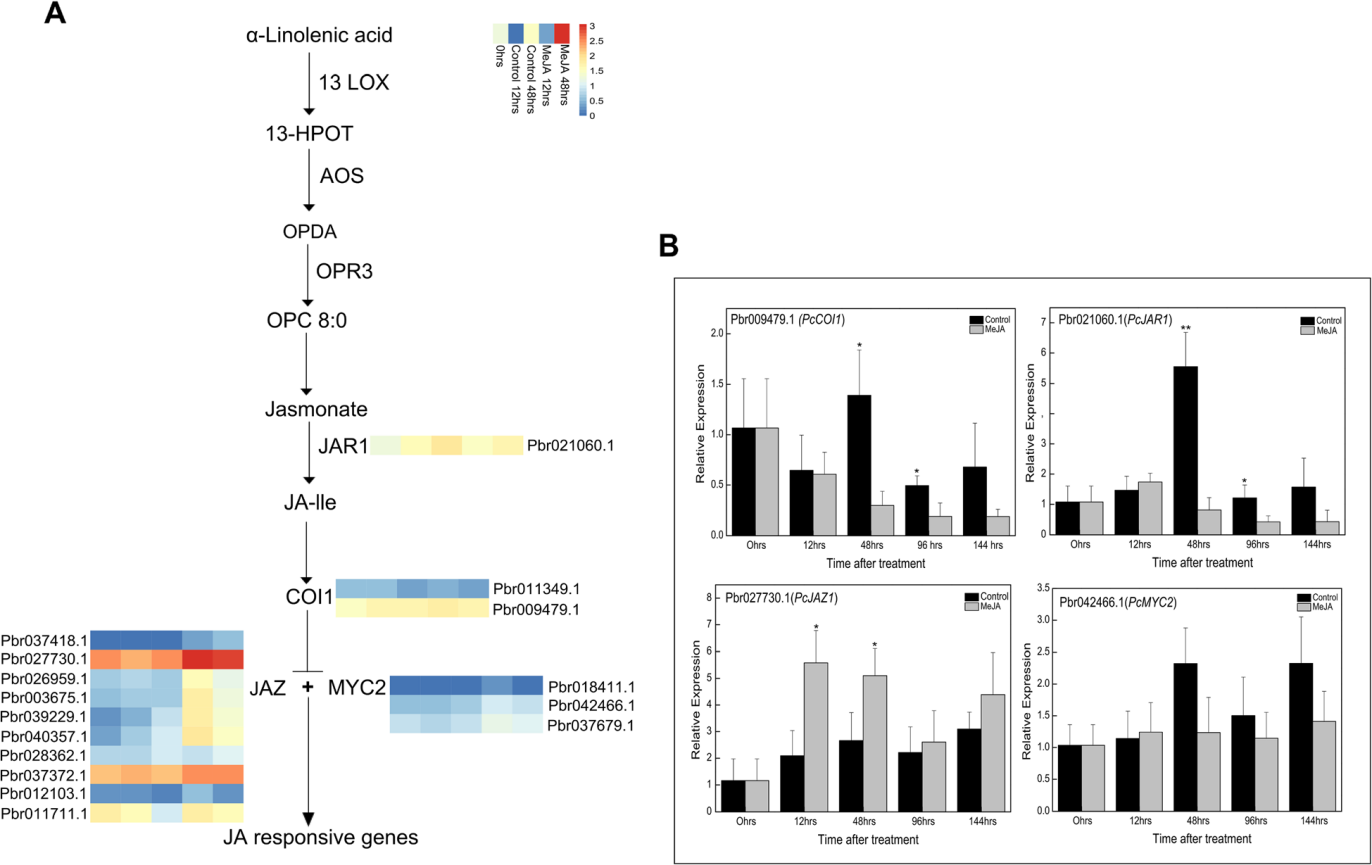
Jasmonate signal transduction pathway. **a** Transcriptional profiles of DEGs associated with the JA signaling pathway. The log_10_ (FPKM + 1) values for the DEGs were calculated based on three biological replicates of pear calli for each time-point. The progression of the color scale from blue to red represents an increase in the FPKM values. **b** Verification of the expression of differentially expressed genes in the JA pathway by qRT-PCR analysis

Several jasmonate signaling factors were identified after annotating DEGs associated with the jasmonate signal transduction pathway (Fig. 3a). For example, Pbr021060.1 was differentially expressed and annotated as *PcJAR1*. The *PcJAR* transcript level was lower in MeJA-treated pear calli than in the untreated control. Two *PcCOI1* genes (Pbr011349.1 and Pbr009479.1) were differentially expressed, with expression levels that were upregulated in response to the MeJA treatment. Furthermore, 10 JAZ genes belonging to the TIFY family had upregulated expression levels. The transcript abundance of the differentially expressed JAZ genes in pear calli was lower at 48 h than at 12 h after the MeJA treatment. Additionally, Pbr018411.1, Pbr042466.1, and Pbr037679.1, which were annotated as *PcMYC2*, were more highly expressed in the control calli than in the MeJA-treated calli. The relative expression of selected genes was further analyzed by quantitative real-time (qRT)-PCR to verify the sequencing data. The expression levels of the selected jasmonate signaling factor genes were consistent with the RNA-seq data (Fig. 3b).

In addition to jasmonate, several DEGs were revealed to be involved in signal transduction pathways related to other plant hormones, including cytokinin, ethylene, auxin, abscisic acid, and brassinosteroid (Additional file 6: Table S3).

### Identification of WGCNA modules associated with flavonoid biosynthesis

**Fig. 4:**
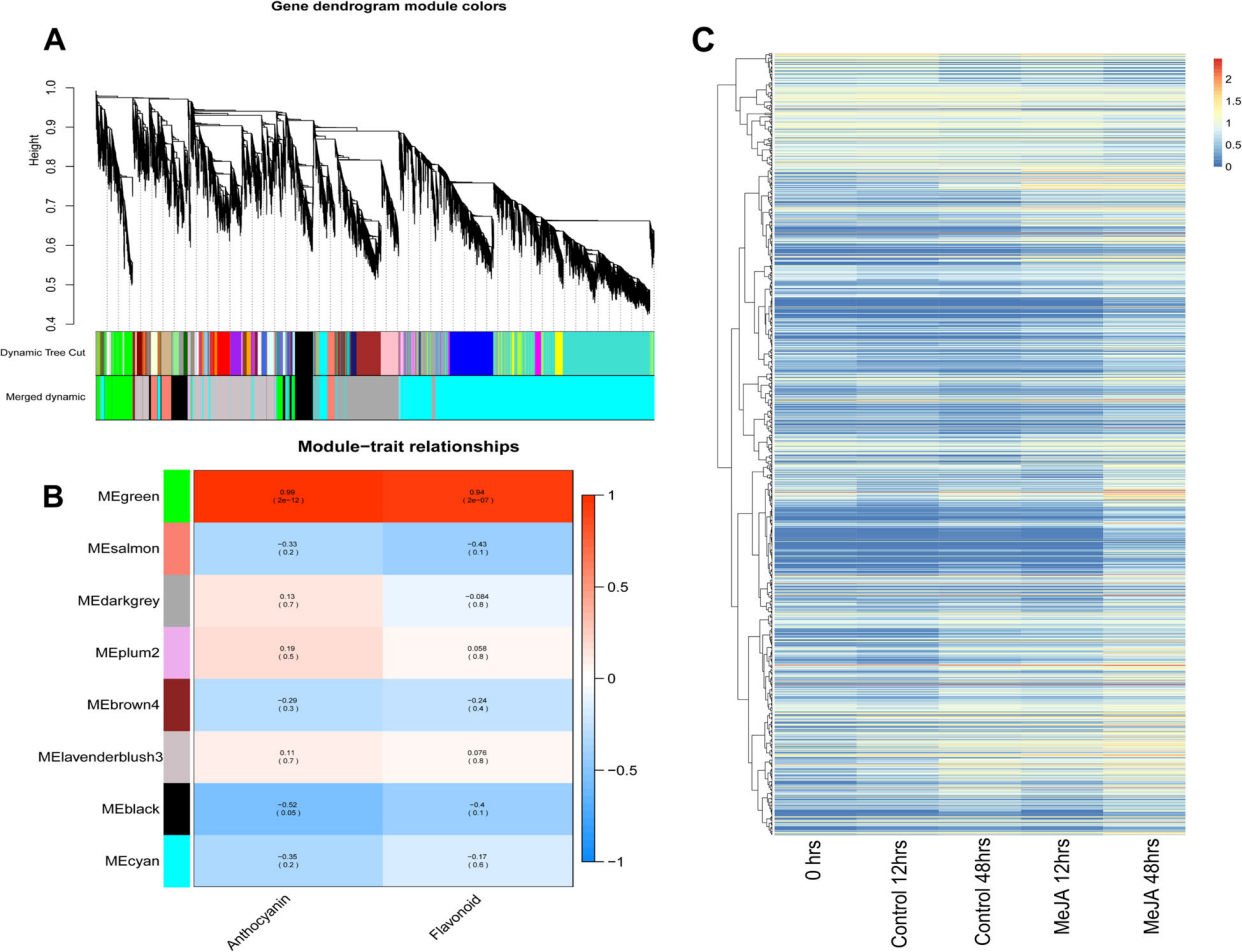
Weighted gene co-expression network analysis of the DEGs identified in the MeJA-treated pear calli. **a** Dendrogram with co-expressed gene modules. **b** Module–trait correlations and *p*-values (in parentheses). The color scale on the right presents the module–trait correlations from − 1 (blue) to 1 (red). The Anthocyanin and Flavonoid panels represent anthocyanin biosynthesis and flavonoid biosynthesis as traits. **c** Heat map presenting the expression patterns of the DEGs in the ME “green” module. Clustering applied the log_10_ (FPKM + 1) value. Red and blue denote genes with high and low expression levels, respectively

A gene co-expression network was constructed via a weighted gene co-expression network analysis (WGCNA) to identify the DEGs associated with MeJA-induced flavonoid biosynthesis. The individual branches of the dendrogram represent the clusters of interconnected genes (i.e., modules). Hierarchical clustering identified eight co-expressed WGCNA modules (Fig. 4a). Each module was analyzed regarding their co-expression related to the trait phenotype (anthocyanin and flavonoid contents). The largest (3139 genes) and smallest (77 genes) modules were “lavenderblush” and “plum”, respectively. An analysis of the module–trait relationships revealed that the “green” module was highly positively correlated with the pear calli anthocyanin (*r* = 0.99, *p* = 2 × 10^− 12^) and flavonoid (*r* = 0.94, *p* = 2 × 10^− 7^) contents (Fig. 4b). The “green” module comprised 1334 genes, and their overall expression patterns are presented in Fig. 4c. This module was selected for further analyses because it was the module most positively correlated with flavonoid biosynthesis in pear calli.

### Flavonoid biosynthesis pathway

**Fig. 5:**
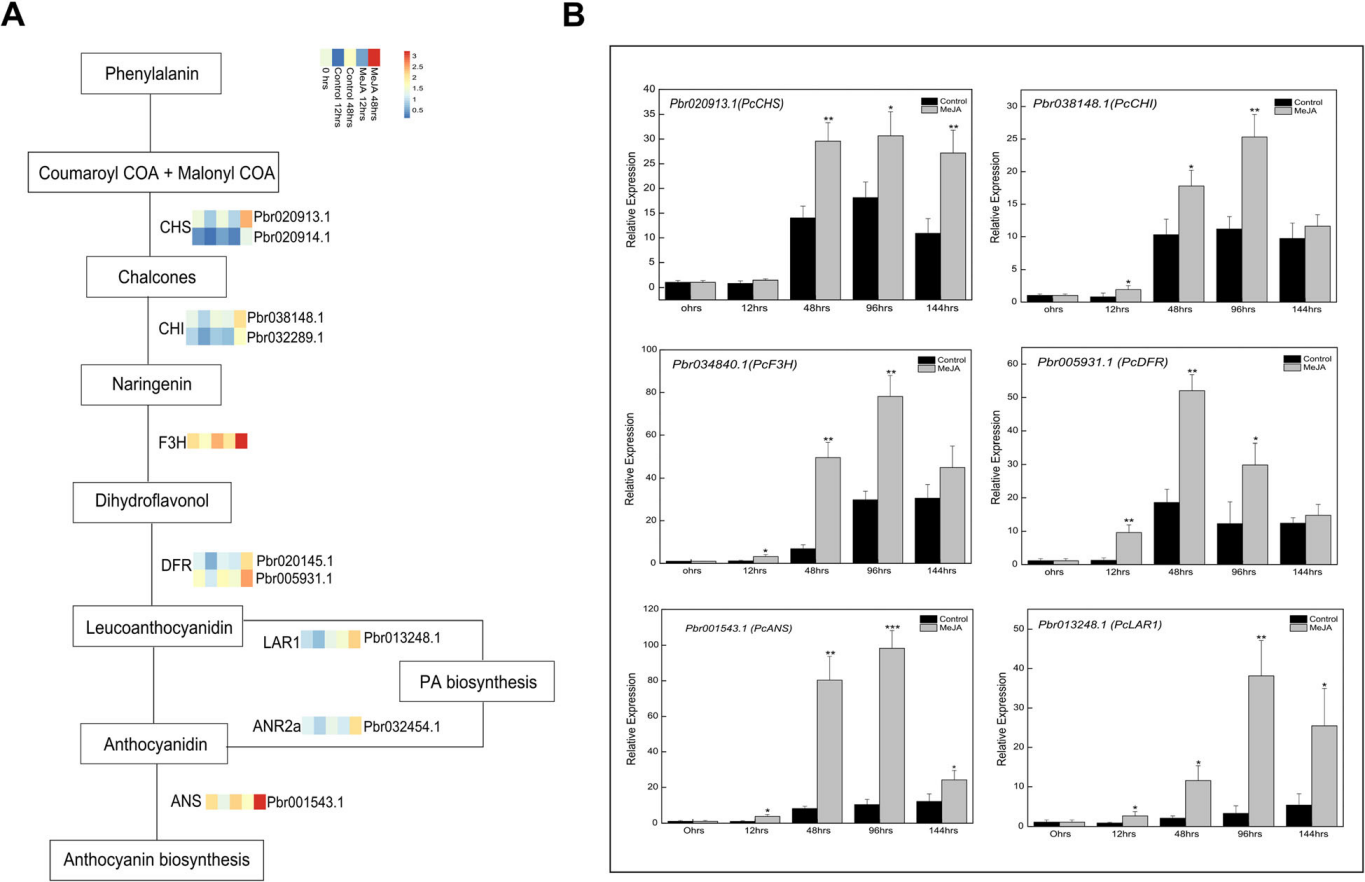
Flavonoid biosynthesis pathway. **a** Transcriptional profiles of differentially expressed structural genes in the flavonoid biosynthesis pathway. The log_10_ (FPKM + 1) values for the DEGs were calculated based on three biological replicates of pear calli for each time-point. The progression of the color scale from blue to red represents an increase in the FPKM values. **b** Verification of the expression of differentially expressed structural genes in the flavonoid biosynthesis pathway by qRT-PCR analysis

Several structural genes were identified after identifying the DEGs related to the flavonoid biosynthesis pathway. For example, *PcCHS* (Pbr020913.1 and Pbr020914.1), *PcCHI* (Pbr038148.1 and Pbr032289.1), and *PcF3H* (Pbr034840.1) were identified as EBGs. Additionally, Pbr020145.1 and Pbr005931.1 were annotated as *PcDFR*. The Pbr001543.1 (*PcANS*) gene was identified as specifically involved in anthocyanin biosynthesis, whereas Pbr013248.1 (*PcLAR1*) and Pbr032454.1 (*PcANR2a*) were revealed to affect proanthocyanidin biosynthesis. We mapped the selected structural genes of the flavonoid biosynthesis pathway and determined their expression patterns (Fig. 5a). The EBGs and LBGs were positively correlated with anthocyanin and flavonoid accumulation in pear calli, with expression levels that were significantly upregulated in the MeJA-treated pear calli, especially at 48 h after the treatment. The relative expression levels of selected structural genes were further analyzed by qRT-PCR to verify the sequencing data. The results indicated *PcDFR* expression was approximately 2-fold higher in MeJA-treated pear calli than in the control calli at 48 h after the treatment. The *PcANS* expression level was upregulated by the MeJA treatment to about 7-fold higher than that in the control calli (Fig. 5b).

### Transcriptional regulation of MeJA-induced flavonoid biosynthesis

Transcription factor families potentially involved in MeJA-induced flavonoid accumulation were identified through the WGCNA (Table 1). The MYB family members were the predominant transcription factor genes regulating flavonoid biosynthesis, followed by the bHLH and AP2-EREBP genes. Additionally, NAC and WRKY family genes were also differentially expressed. Moreover, the TIFY and zinc finger protein (C2H2, C3H, and C2C2-Dof) transcription factor families were also identified as related to the jasmonate signal transduction pathway. A total of 108 differentially expressed transcription factor genes were included in the “green” module following the WGCNA. The transcriptional profiles of differentially expressed transcription factor genes in the “green” module are presented in Additional file 3: Fig. S3. Most of these genes were highly expressed in response to the MeJA treatment. In addition to the MYB transcription factors, the bHLH genes, such as Pbr006544.1, Pbr017127.1, Pbr017379.1, and Pbr030521.1, exhibited significantly upregulated expression in the MeJA-treated calli relative to the control levels. Moreover, Pbr029330.1, Pbr023747.1, and Pbr008278.1 were highly expressed WRKY transcription factor genes following the MeJA treatment.

**Table 1:** Differentially expressed transcription factors related to flavonoid biosynthesis in pear calli

Type of TF	Number of DEGs	Description
MYB	21	Myb (Myeloblastosis) -related protein
bHLH	8	Basic helix-loop-helix protein
NAC	4	NAC domain-containing protein
WRKY	5	WRKY transcription factor
AP2-EREBP	9	Ethylene-responsive transcription factor
HB	6	Homeobox-leucine zipper protein
MADS	3	MADS-box protein
TIFY	5	TIFY protein
C2H2	6	Zinc finger C2H2 domain-containing protein
C3H	4	Zinc finger CCCH domain-containing protein
C2C2-Dof	3	Zinc finger protein
Trihelix	4	Trihelix transcription factor
GNAT	2	GCN5-Related N-Acetyltransferases protein
bZIP	2	Basic Leucine Zipper Domain transcription factor
SNF2	4	SNF2 domain-containing protein
GRAS	2	Scarecrow-like protein
HSF	2	Heat stress transcription factor
Orphans	3	Orphan Transcription Factor
LOB	2	LOB domain-containing protein
Other TFs	11	
Total TFs	108	

### Role of the MYB family in the transcriptional regulation of MeJA-induced flavonoid biosynthesis in pear calli

**Fig. 6:**
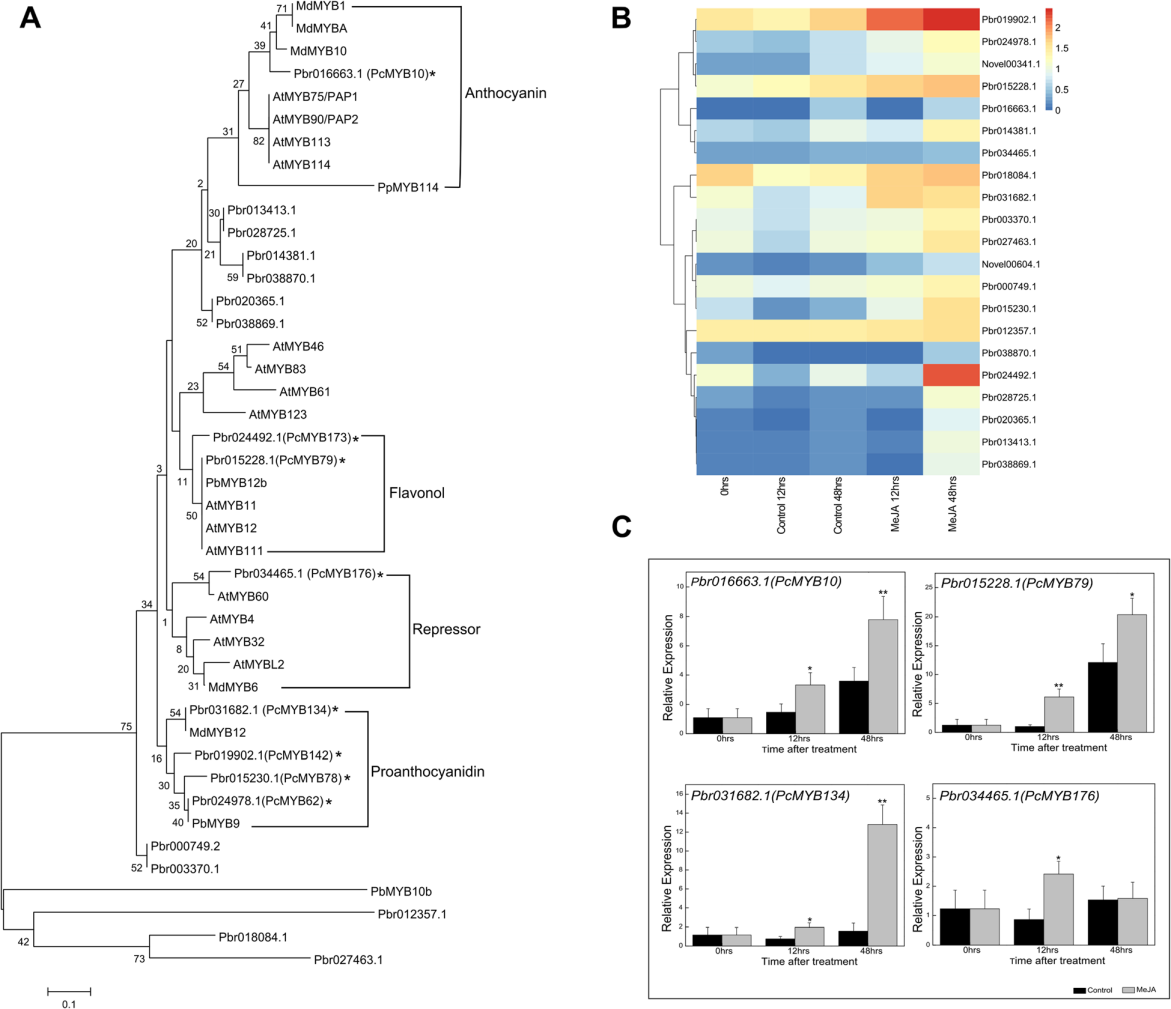
Analysis of differentially expressed MYB transcription factor genes related to flavonoid biosynthesis. **a** Phylogenetic tree with flavonoid regulatory MYB transcription factors in pear (*Pyrus* spp.), apple (*Malus domestica*), and Arabidopsis (*Arabidopsis thaliana*). **b** Heat map presenting the expression patterns of differentially expressed MYB transcription factor genes in the ME “green” module of the weighted gene co-expression network. The progression of the color scale from blue to red represents an increase in the FPKM values. **c** Verification of the expression of differentially expressed MYB transcription factor genes by qRT-PCR analysis

We identified 21 “green” module genes encoding candidate MYB transcription factors involved in MeJA-induced flavonoid biosynthesis in pear calli. A phylogenetic tree was constructed based on known flavonoid regulatory MYB transcription factors in pear (*Pyrus* spp.), apple (*M. domestica*), and Arabidopsis (Fig. 6a). For example, Pbr016663.1 (MYB10) was identified as a known anthocyanin regulatory MYB transcription factor in pear. Additionally, several novel candidate MYB transcription factors were identified. Both Pbr015228.1 (PcMYB79) and Pbr024492.1 (PcMYB173) were phylogenetically related to flavonol regulators such as PbMYB12b in pear and flavonol regulatory MYB transcription factors in Arabidopsis (AtMYB12, AtMYB111, and AtMYB11). Furthermore, four candidate MYB transcription factors [Pbr031682.1 (PcMYB134), Pbr019902.1 (PcMYB142), Pbr015230.1 (PcMYB78), and Pbr024978.1 (PcMYB62)] were identified as potential regulators of proanthocyanidin biosynthesis. In contrast, Pbr034465.1 (PcMYB176) was revealed as a potential repressor because it was grouped with flavonoid biosynthesis repressors in apple and Arabidopsis.

The transcriptional profiles of the candidate MYB transcription factor genes indicated that most of these genes were more highly expressed in the MeJA-treated pear calli than in the control calli, with expression levels that were positively correlated with anthocyanin and flavonoid biosynthesis (Fig. 6b). The relative expression levels of selected MYB transcription factor genes determined by qRT-PCR were consistent with the FPKM values based on the sequencing data (Fig. 6c).

### PcMYB10 and PcMYC2 interact with JAZ repressors

**Fig. 7:**
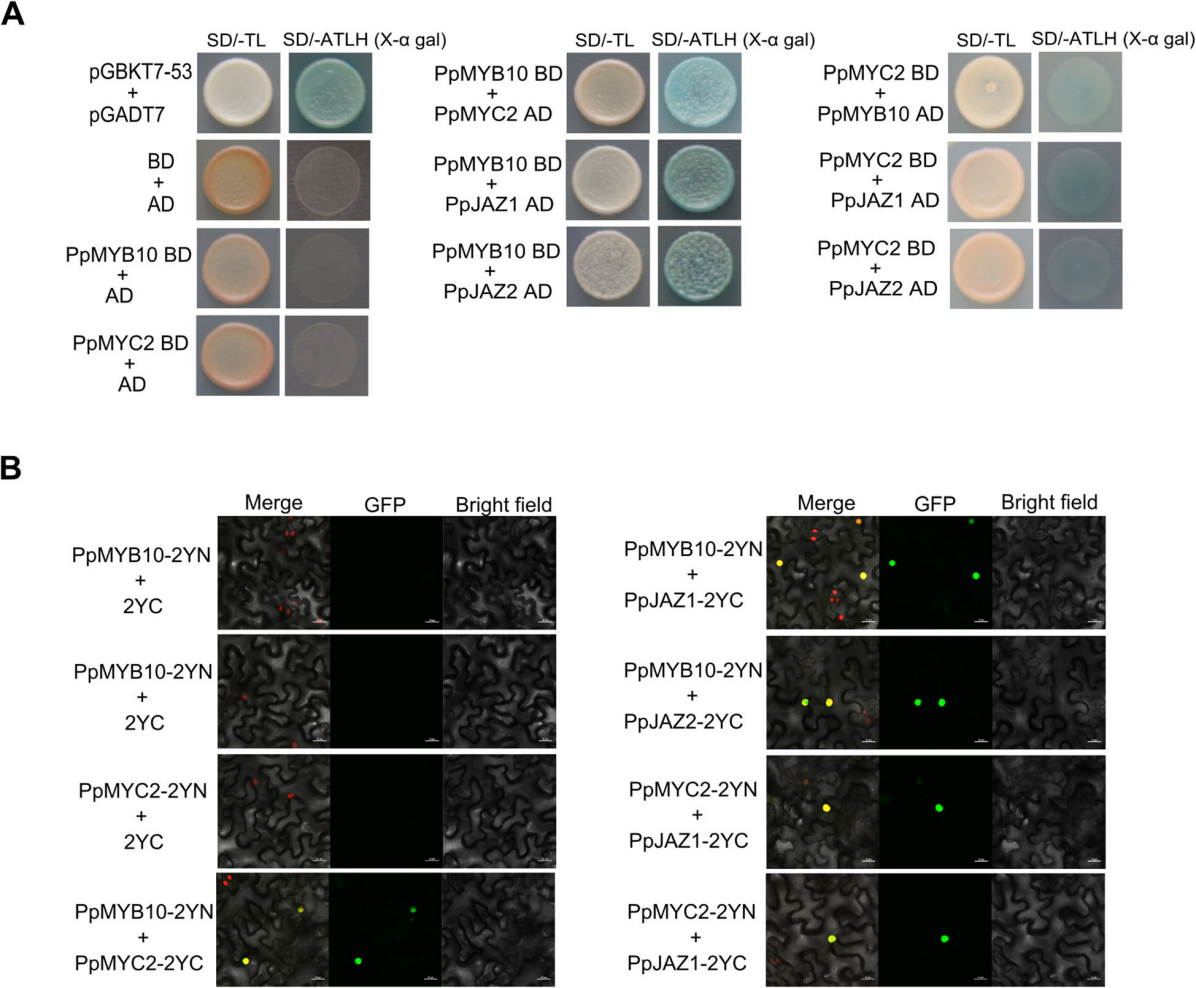
Interactions between PcMYB10 and JA signaling factors. **a** Yeast two-hybrid analyses of the interactions of PcMYB10 with PcMYC2, PcJAZ1, and PcJAZ2. **b** Bimolecular fluorescence complementation assay presenting the interaction of PcMYB10 with PcMYC2, PcJAZ1, and PcJAZ2 in *Nicotiana benthamiana* leaves

The physical interactions of PcMYB10 and PcMYC2 with selected JAZ proteins were analyzed in yeast two-hybrid (Y2H) assays. The results demonstrated that PcMYB10 and PpMYC2 can physically interact with each other and with PcJAZ1 as well as PcJAZ2 (Fig. 7a). These interactions were verified in bimolecular fluorescence complementation (BiFC) assays, in which fluorescence was undetectable in the negative controls (Fig. 7b). However, consistent with the Y2H results, a strong green fluorescent protein signal was observed in the nuclei when PcMYB10-2YN was co-expressed with PcMYC2-2YC, PcJAZ1-2YC, and PcJAZ2-2YC. Additionally, fluorescence was detected in samples co-infiltrated with PcMYC2-2YN and PcJAZ1-2YC as well as PcJAZ2-2YC. These findings indicate that PcMYB10 and PcMYC2 can physically interact with each other and with PcJAZ1 and PcJAZ2.

## Discussion

### MeJA induces flavonoid biosynthesis in pear calli

In plants, jasmonates are essential signaling molecules [37] that promote the biosynthesis of secondary metabolites, especially flavonoids [38]. Flavonoids are important determinants of fruit quality and economic value because of their effects on color, aroma, astringency, and antioxidant properties [39]. Therefore, over the last few decades, numerous studies have been performed to develop strategies to increase fruit flavonoid contents via jasmonate treatments [22, 27, 30, 31]. In the present study, the application of exogenous MeJA activated the jasmonate signaling pathway in pear calli. The expression levels of many DEGs were upregulated in the MeJA-treated pear calli relative to the corresponding levels in the untreated control (Additional file 1: Fig. S1). Additionally, several important jasmonate signaling factors (JAR1, COI1, JAZ, and MYC2) were annotated as part of the jasmonate signal transduction pathway (Fig. 3).

We used pear calli to study the effects of MeJA on flavonoid biosynthesis because they can be continuously and uniformly produced to efficiently use the available space. We observed that MeJA significantly enhanced the biosynthesis of flavonoids in pear calli, especially anthocyanin (Fig. 1). Similarly, previous studies concluded that MeJA promotes anthocyanin and proanthocyanidin accumulation in apple calli [18, 20, 33]. Furthermore, efficient in vitro systems reportedly can produce high quality anthocyanins on a commercial scale [40].

In this study, the MeJA treatment upregulated the expression of EBGs, such as *PcCHS*, *PcCHI*, and *PcF3H*, which are involved in the early stages of the flavonoid biosynthesis pathway. Additionally, the LBGs, which are specifically involved in anthocyanin and proanthocyanidin biosynthesis, were more highly expressed in the MeJA-treated pear calli than in the control calli (Fig. 5). Both DFR and ANS are considered key enzymes for anthocyanin biosynthesis [9]. Consistent with our results, Shan et al. [17] reported that jasmonate strongly upregulates the expression of *AtDFR* in Arabidopsis seedlings, thereby regulating anthocyanin accumulation. Furthermore, the expression levels of other flavonoid biosynthetic genes, including *AtPAL*, *AtCHS*, *AtCHI*, *AtF3H*, and *AtF3′H*, also increased in response to jasmonate, although the expression levels were still relatively low. Sun et al. [20] demonstrated that the application of exogenous MeJA enhances the anthocyanin accumulation in red-fleshed apple calli because of the associated upregulated *MdCHS*, *MdF3H*, and *MdUFGT* expression*.* In addition to anthocyanin biosynthesis-related genes, the expression of key genes involved in proanthocyanidin biosynthesis (*PcANR2a* and *PcLAR1*) was also upregulated by MeJA (Fig. 5). Moreover, a KEGG analysis revealed that the flavonoid biosynthesis pathway was significantly enhanced in the MeJA-treated pear calli compared with the untreated control (Fig. 2).

### Transcriptional regulation of MeJA-mediated flavonoid biosynthesis in pear calli based on RNA sequencing data

Transcription factors regulate the expression of flavonoid biosynthesis pathway structural genes. For example, MYB, bHLH, and WDR proteins form the MBW complex that regulates flavonoid biosynthesis in many plant species [12]. Previous studies indicated that MYB transcription factors are the key elements in the regulatory networks controlling specific gene expression patterns during flavonoid biosynthesis [1]. In pear, PpMYB10 was initially identified as a R2R3-MYB transcription factor that positively regulates anthocyanin biosynthesis [41]. Additionally, PbMYB10b and PbMYB9 were characterized as positive regulators of anthocyanin and proanthocyanidin biosynthesis in pear [42]. Earlier investigations determined that PpMYB114 and PpbHLH3 can co-regulate anthocyanin biosynthesis in pear fruit [43], whereas PbMYB12b was functionally annotated as a flavonol regulator in pear [44]. However, MYB transcription factors involved in jasmonate-mediated flavonoid biosynthesis have not been specifically characterized in pear. Nevertheless, in apple, MdMYB9 and MdMYB11 reportedly interact with MdbHLH3 and MdTTG1 to form a MBW complex that regulates jasmonate-mediated anthocyanin and proanthocyanidin accumulation [33]. Recently, *MdMYB24L* was overexpressed in apple calli and functionally characterized as a gene encoding a jasmonate-responsive MYB transcription factor contributing to MeJA-induced anthocyanin accumulation in apple. Other studies proved that jasmonate-induced anthocyanin accumulation in Arabidopsis is mediated by MYB transcription factors, including PAP1 (MYB75), PAP2 (MYB90), and GL3, that upregulate the expression of anthocyanin biosynthetic genes [17, 32].

RNA sequencing is an effective tool for clarifying the transcriptional regulation of essential genes in secondary metabolite biosynthesis pathways [45]. On the basis of a WGCNA, we identified 21 candidate MYB transcription factor genes whose expression levels were significantly positively correlated with MeJA-induced flavonoid biosynthesis in pear calli. These genes included Pbr016663.1 (PcMYB10), which encodes a known MYB transcription factor that positively regulates anthocyanin biosynthesis in pear. Interestingly, we detected several novel MYB candidates as potential regulators of proanthocyanidin and flavonol biosynthesis in pear (Fig. 6).

Genes encoding other candidate transcription factors belonging to bHLH, AP2-EREBP, NAC, WRKY, and TIFY families were also revealed as differentially expressed based on our transcriptome data (Additional file 3: Fig. S3). Several bHLH transcription factors that regulate jasmonate-responsive anthocyanin accumulation in plants have been reported. A previous study proved that MYC2, which belongs to the bHLH family, regulates diverse jasmonate responses in Arabidopsis, including anthocyanin biosynthesis, wound responses, root growth inhibition, and oxidative stress adaptations [17]. Additionally, MYC3 and MYC4, which are close homologs of MYC2, function additively with MYC2 in the jasmonate signaling pathway [46]. Moreover, GL3, EGL3, and TT8 bHLH transcription factors are also positive regulators of jasmonate-responsive anthocyanin accumulation in Arabidopsis [32]. In apple, MdMYC2 has been functionally characterized as a positive regulator of jasmonate-induced anthocyanin biosynthesis [47]. However, bHLH transcription factors involved in the pear jasmonate-mediated flavonoid biosynthesis have yet to be reported. In the current study, we identified several candidate bHLH transcription factors, including PcMYC2, that might participate in the jasmonate-induced flavonoid biosynthesis in pear calli (Fig. 3 and Additional file 3: Fig. S3).

### Molecular mechanism associated with MeJA-induced flavonoid biosynthesis in pear calli

The molecular mechanism underlying jasmonate-induced anthocyanin biosynthesis has been thoroughly characterized in Arabidopsis [17, 32, 35, 48]. The JAZ proteins are believed to repress the jasmonate signaling pathway. The JAZ repressors directly interact with MYB transcription factors (PAP1, PAP2, and GL1) and bHLH transcription factors (GL3, EGL3, and TT8) of the MBW complex to inhibit transcription and subsequently repress anthocyanin biosynthesis in Arabidopsis. Following the jasmonate signal-induced degradation of JAZ proteins, the MBW complex is activated to induce the expression of the downstream structural genes and mediate jasmonate-induced anthocyanin biosynthesis [32]. Moreover, Shan et al. [17] revealed that the F-box protein COI1 is essential for the expression of transcription factor genes, including those encoding PAP1, PAP2, and GL3. In apple, an earlier study concluded that MdJAZ2 inhibits the recruitment of MdbHLH3 to the *MdMYB9* and *MdMYB1* promoters. After jasmonate signals are perceived, MdbHLH3 is released to form the MBW complex involved in activating the downstream genes related to flavonoid biosynthesis [33]. However, the molecular mechanism associated with jasmonate-mediated flavonoid biosynthesis remains largely unknown in pear.

In the present study, we observed that PcMYB10 and PcMYC2 can directly interact with each other and with JAZ proteins (PcJAZ1 and PcJAZ2) (Fig. 7). In contrast, our transcriptome data indicated that 10 JAZ genes were more highly expressed in the MeJA-treated calli than in the control calli (Fig. 3). Although the transcription of JAZ genes may be upregulated, it may not be consistent with the high translated protein levels because of post-translational regulatory activities [49]. In a previous study on apple, MdJAZ8 and MdJAZ11 were observed to form a complex with the MdMYB24-like protein to weaken the transcriptional activity of the MYB–MYC2 complex, and in response to a jasmonate application, MdJAZ8 and MdJAZ11 were degraded to release MdMYC2 and MdMYB24L, thereby promoting JA-mediated anthocyanin accumulation [18]. Consequently, the findings of the present suggest that the PcMYB10–PcMYC2 molecular complex may be involved in the transcriptional regulation of jasmonate-mediated flavonoid biosynthesis in pear. However, this will need to be experimentally confirmed in future studies.

## Conclusions

In the current study, the application of exogenous MeJA activated the jasmonate signaling pathway. Moreover, MeJA induced the accumulation of flavonoids, especially anthocyanin, in pear calli by upregulating the expression of structural genes (*PcCHS*, *PcCHI*, *PcF3H*, *PcDFR*, *PcANS*, and *PcLAR1*) in the flavonoid biosynthesis pathway. The MYB family was prominently involved in the transcriptional regulation of flavonoid biosynthesis, with the bHLH, AP2-EREBP, NAC, WRKY, and TIFY family members also contributing. Additionally, protein interaction assays suggested the PcMYB10–PcMYC2 molecular complex might influence the transcriptional regulation of jasmonate-mediated flavonoid biosynthesis in pear calli. Our comprehensive transcriptome analysis revealed a set of candidate transcription factors that may be relevant for future functional studies related to the transcriptional regulation of MeJA-mediated flavonoid biosynthesis in pear.

## Methods

### Plant materials and treatment

Pear calli were induced from the flesh of young ‘Clapp’s Favorite’ (*Pyrus communis*) pear fruit in our in vitro laboratory according to a published protocol [50]. The pear fruit sample was kindly provided by Prof. Yuanjun Li from the Yantai Academy of Agricultural Sciences, Yantai, Shandong, China. Briefly, pear calli were grown in darkness on MS solid medium supplemented with sucrose (30 g/L), 6-benzylaminopurine (0.5 mg/L), and 2,4-dichlorophenoxyacetic acid (1.0 mg/L). The calli were sub-cultured every 3 weeks. They were then transferred to MS medium containing MeJA, which had been diluted to 50 μmol/L in methanol. Accordingly, control calli were transferred to MS medium with 1% methanol. The treated and control calli were incubated under continuous white light (2000 lx) at 24 °C. Calli samples were collected at 0, 12, 24, 48, 96, and 144 h after the treatment. At each time-point, samples were collected from three calli plates (three replicates). The experiment was conducted according to a completely randomized design. The collected calli samples were immediately frozen in liquid nitrogen and stored at − 80 °C until analyzed.

### Determination of the total flavonoid content

Flavonoids were analyzed with the Cominbio Plant Flavonoid Extraction kit (Suzhou Keming Biotechnology Co., Ltd., Suzhou, China). Specifically, flavonoids were extracted with 60% ethanol and then complexed with an aluminum ion in an alkaline nitrite solution. The absorbance (at 530 nm) of the sample extract was measured with the DU800 spectrophotometer (Beckman Coulter, Brea, CA, USA), after which the flavonoid content was calculated with the following formula:

$$ \mathrm{Flavonoid}\ \mathrm{content}\ \left[\mathrm{mg}/\mathrm{g}\ \mathrm{fresh}\ \mathrm{weight}\ \left(\mathrm{FW}\right)\right]=0.797\times \left({\Delta \mathrm{A}}_{530}-\kern0.5em 0.0007\right)/\mathrm{FW}. $$

### Determination of the total anthocyanin content

Anthocyanins were extracted from the pear calli as previously described [14]. Briefly, 0.2 g frozen pear calli were treated with 1 mL methanol:acetic acid (99:1 volume) overnight in darkness at 4 °C. The absorbance (at 530, 620, and 650 nm) was measured with the DU800 spectrophotometer. The total anthocyanin content was calculated with the following formula:

$$ \mathrm{Anthocyanin}\ \mathrm{content}\ \left(\mathrm{mg}/100\;\mathrm{g}\ \mathrm{FW}\right)=\left[\left({\mathrm{A}}_{530}\hbox{-} {\mathrm{A}}_{650}\right)\hbox{-} 0.2\times \left({\mathrm{A}}_{650}\hbox{-} {\mathrm{A}}_{620}\right)\right]/\mathrm{FW}. $$

### RNA extraction and qRT-PCR analysis

Total RNA was extracted from the collected samples according to the cetyltrimethylammonium bromide method [51]. The qRT-PCR analysis was performed as previously described [52]. Details regarding the qRT-PCR primers are listed in Additional file 7: Table S4.

### RNA-seq analysis

The pear calli sampled at 0, 12, and 48 h after the MeJA treatment as well as the corresponding control samples (1% methanol) were used for the RNA-seq analysis, which was completed as described by Bai et al. [50]. The libraries were prepared and then sequenced with the HiSeq X system (Illumina, San Diego, CA, USA) by Novogene (Beijing, China). The clean reads were mapped to the *Pyrus bretschneideri* genome sequence (http://gigadb.org/dataset/100083) with the default parameters of HISAT2.

### GO and KEGG enrichment analyses of differentially expressed genes

The GO enrichment analysis of DEGs was completed with the goseq R package, in which the gene length bias was corrected. The GO terms with a corrected *p*-value less than 0.05 were considered significantly enriched. The KOBAS software was used to identify the significantly enriched KEGG pathways among the DEGs (http://www.genome.jp/kegg/) [53].

### Construction and visualization of co-expression modules

A WGCNA was performed with the R package according to a published method [54]. The sequences of flavonoid regulatory MYB transcription factors in pear (*Pyrus* spp.), apple (*M. domestica*), and Arabidopsis were downloaded from the NCBI database. Protein accessions are provided in Additional file 8: Table S5. A phylogenetic tree was generated with the neighbor-joining method (500 bootstrap replicates) of the MEGA 6.0 program.

### Yeast two-hybrid assay

Yeast two-hybrid assays were performed with the Matchmaker™ Gold Yeast Two-Hybrid System Kit (TaKaRa, Dalian, China). The full-length coding sequences encoding the prey and bait proteins were cloned into the pGADT7 (AD) and pGBKT7 (BD) vectors, respectively. First, the full-length PcMYB10-BD and PcMYC2-BD plasmids were inserted into Y2HGold cells with an empty AD vector, after which a lack of self-activation was confirmed for PcMYB10-BD and PcMYC2-BD. Y2HGold competent cells were co-transformed with the recombinant gene-AD and gene-BD plasmids and spread agar-solidified SD/−Leu/−Trp medium. To evaluate potential physical interactions, the co-transformed colonies were selected on SD medium lacking adenine, histidine, leucine, and tryptophan, supplemented with X-α-gal.

### Bimolecular fluorescence complementation assay

The BiFC assays were conducted as previously described [52]. Briefly, the *PcMYB10*, *PcMYC2*, *PcJAZ1*, and *PcJAZ2* coding sequences without terminator codons were amplified and cloned into the p2YN and p2YC vectors. The resulting recombinant plasmids were inserted into *Agrobacterium tumefaciens* strain GV3101 cells, which were then infiltrated into *Nicotiana benthamiana* leaves. At 48 h after the infiltration, fluorescence was detected in the transformed leaves with a confocal laser scanning microscope (Nikon, Japan).

### Phylogenetic analysis

A phylogenetic tree was generated with the neighbor-joining method of the MEGA 6.0 program. The tree included bootstrap values from 1000 replications next to the branch nodes and a bar indicating an evolutionary distance of 0.1%. The accession numbers of the flavonoid regulatory MYB transcription factors in pear (*Pyrus* spp.), apple (*M. domestica*), and Arabidopsis that were included in the phylogenetic analysis are listed in Additional file 8: Table S5.

### Statistical analysis

Experiments were performed according to a completely randomized design. Significant differences (*p < 0.05, **p < 0.01, and ***p < 0.001) between two independent treatments were determined with Student’s *t*-test. All data were analyzed with the SPSS software (version 25) (SPSS Inc., Chicago, IL, USA).

## Supplementary information

**Additional file 1: Figure S1.** Volcano plots for differentially expressed genes between the MeJA-treated pear calli and the control calli (1% methanol) at different time-points.

**Additional file 2: Figure S2**. Gene ontology (GO) classification of differentially expressed upregulated unigenes in the MeJA-treated pear calli after 12 and 48 h. The x-axis and y-axis present the enriched GO terms and the number of differentially expressed genes, respectively.

**Additional file 3: Figure S3.** Heat map presenting the expression patterns of differentially expressed transcription factor genes in the ME “green” module of the weighted gene co-expression network. The progression of the color scale from blue to red represents an increase in the FPKM values.

**Additional file 4: Table S1.** Detail statistics of the sequencing data.

**Additional file 5: Table S2.** Gene expression quantification.

**Additional file 6: Table S3.** Differentially expressed genes involved in plant hormone signal transduction pathways.

**Additional file 7: Table S4.** Primers used for the qRT-PCR analysis.

**Additional file 8: Table S5.** Accession numbers of the flavonoid regulatory MYB transcription factors in pear (*Pyrus* spp.), apple (*Malus domestica*), and Arabidopsis (*Arabidopsis thaliana*) that were included in the phylogenetic analysis.

## Data Availability

The relevant data in this study are included in this article and the supplementary files.

## Abbreviations

MeJA: Methyl jasmonate
DEG: Differentially expressed gene
CHS: Chalcone synthase
CHI: Chalcone isomerase
F3H: Flavanone 3-hydroxylase
DFR: Dihydroflavonol 4-reductase
ANS: Anthocyanin synthase
EBG: Early biosynthetic gene
LBG: Late biosynthetic gene
WGCNA: Weighted gene co-expression network analysis
