# Supplementary figures and images for: Transcriptome analysis provides new insights into the transcriptional regulation of methyl jasmonate-induced flavonoid biosynthesis in pear calli
Source: BMC Plant Biol. 2020 Aug 25;20:388. doi: 10.1186/s12870-020-02606-x (PMC7446162; doi:10.1186/s12870-020-02606-x)

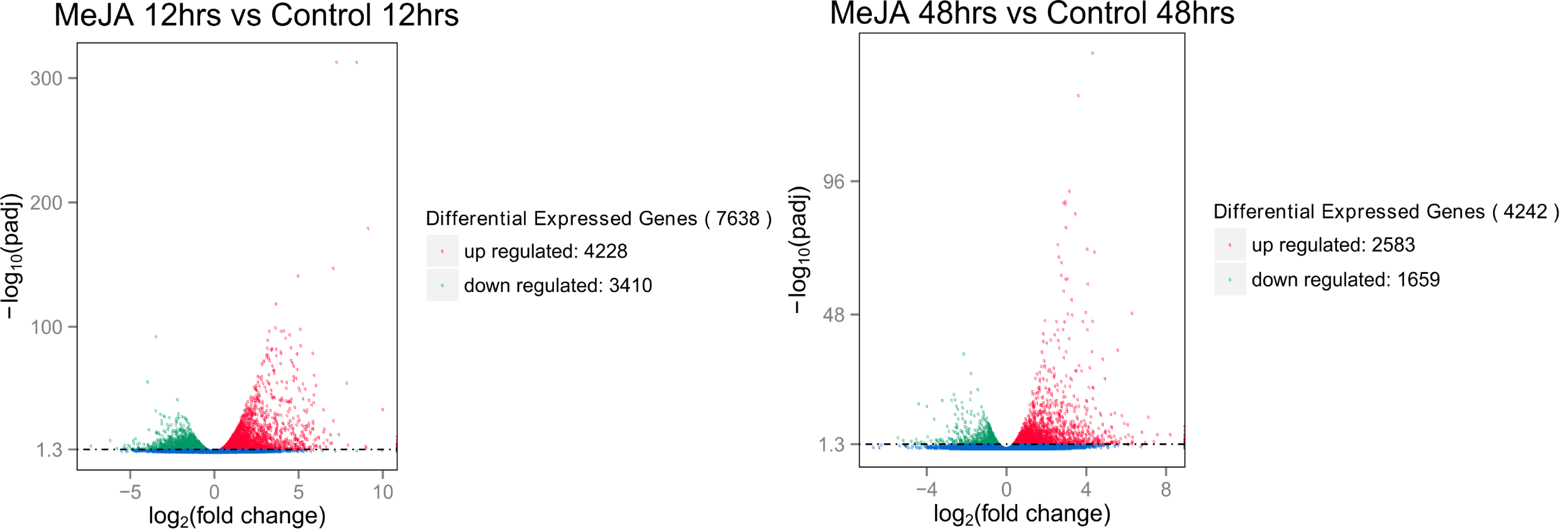

Supplement: Supplementary file 1 — Additional file 1: Figure S1. Volcano plots for differentially expressed genes between the MeJA-treated pear calli and the control calli (1% methanol) at different time-points. [file 12870_2020_2606_MOESM1_ESM.tif]

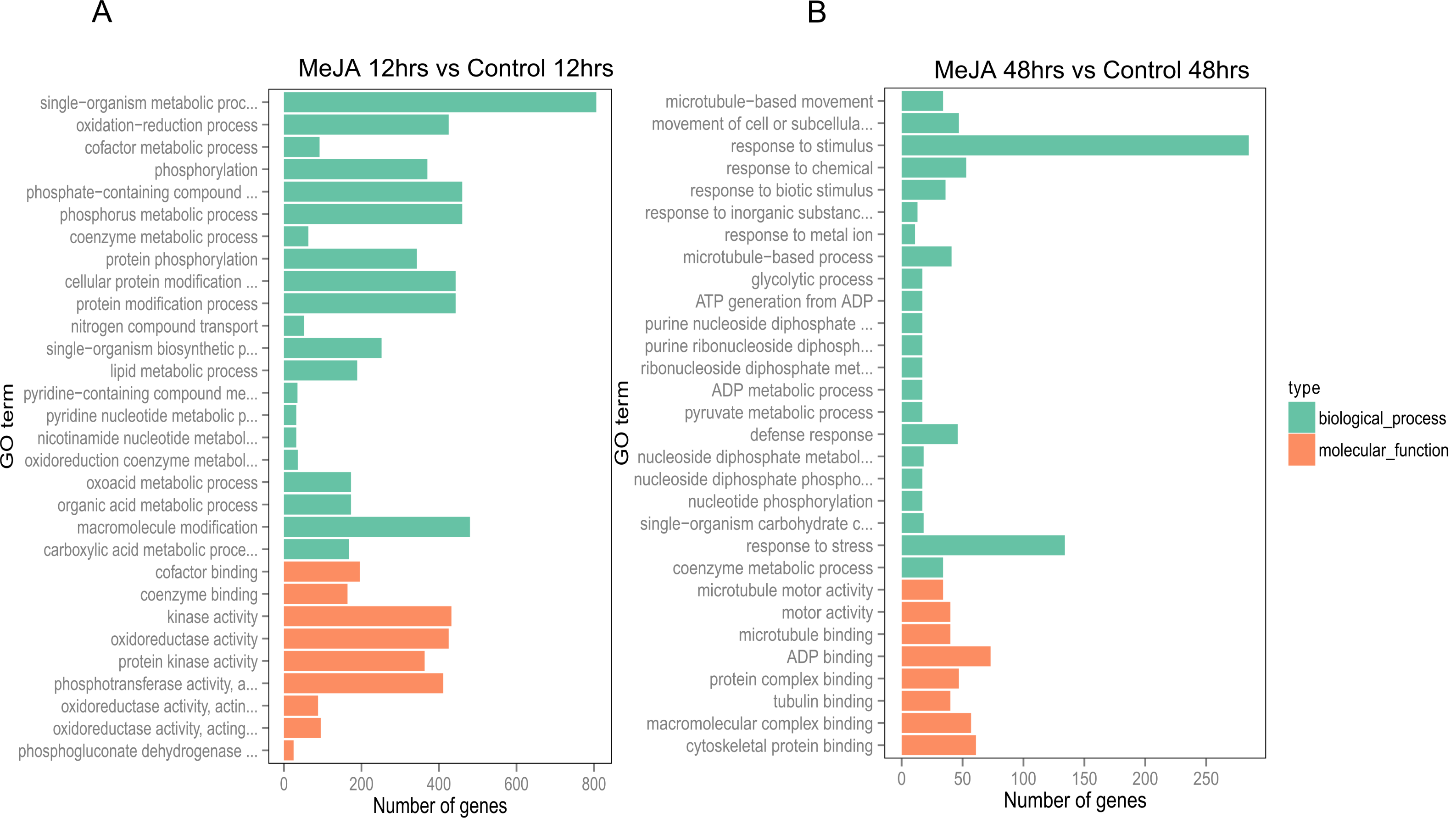

Supplement: Supplementary file 2 — Additional file 2: Figure S2. Gene ontology (GO) classification of differentially expressed upregulated unigenes in the MeJA-treated pear calli after 12 and 48 h. The x-axis and y-axis present the enriched GO terms and the number of differentially expressed genes, respectively. [file 12870_2020_2606_MOESM2_ESM.tif]

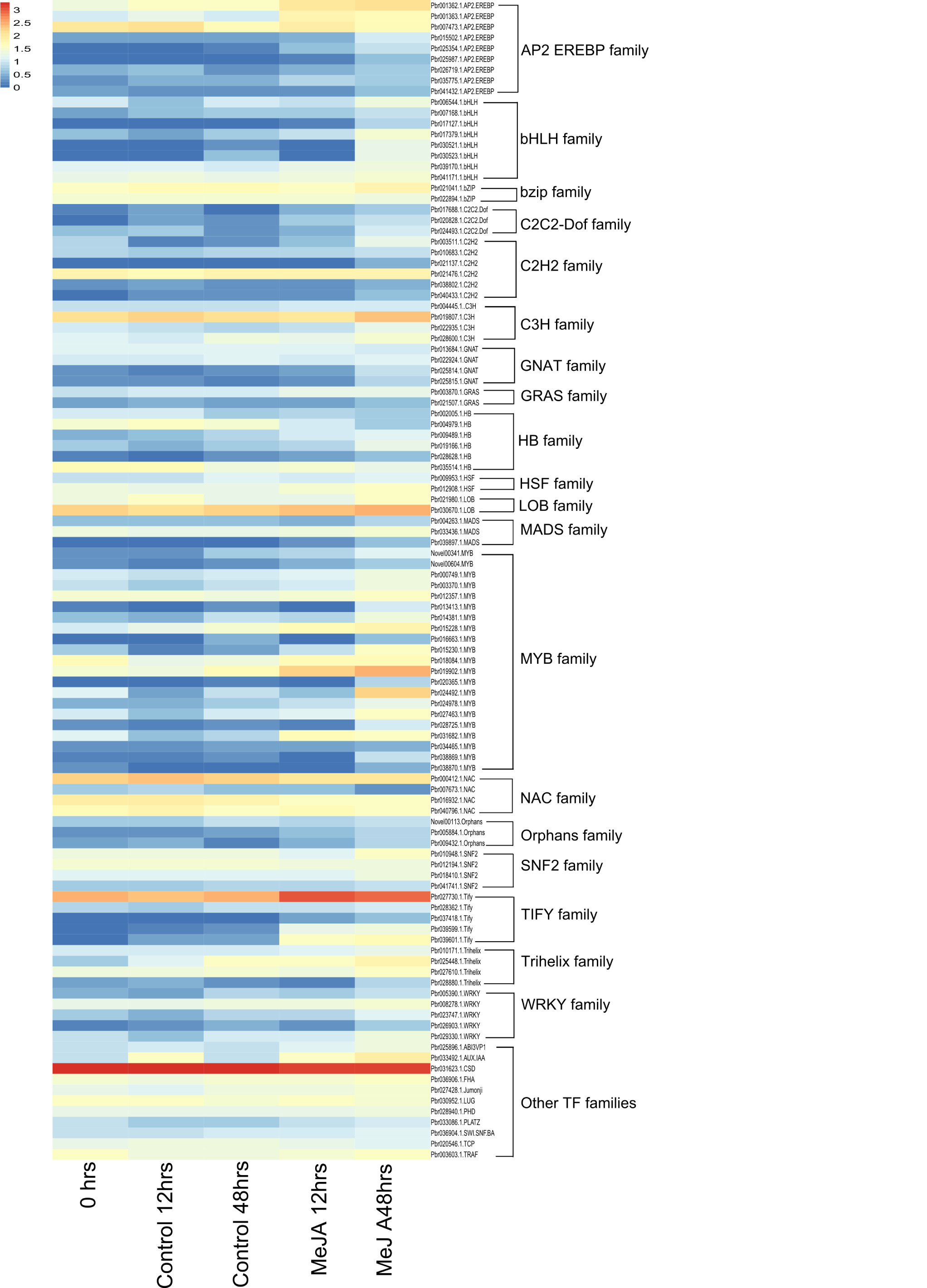

Supplement: Supplementary file 3 — Additional file 3: Figure S3. Heat map presenting the expression patterns of differentially expressed transcription factor genes in the ME “green” module of the weighted gene co-expression network. The progression of the color scale from blue to red represents an increase in the FPKM values. [file 12870_2020_2606_MOESM3_ESM.tif]
