# Supplementary material for: Transcriptome analysis provides new insights into the transcriptional regulation of methyl jasmonate-induced flavonoid biosynthesis in pear calli
Source: BMC Plant Biol. 2020 Aug 25;20:388. doi: 10.1186/s12870-020-02606-x (PMC7446162; doi:10.1186/s12870-020-02606-x)
Supplement: Supplementary file 6 — Additional file 6: Table S3. Differentially expressed genes involved in plant hormone signal transduction pathways. [file 12870_2020_2606_MOESM6_ESM.docx]

**Additional file 6: Table S3. Differentially expressed genes involved in plant hormone signal transduction pathways**

| **Pathway** | **Gene ID** | **Peak orientation** | **Annotation** |
| --- | --- | --- | --- |
| Jasmonic acid | Pbr037418.1 | Up | Protein TIFY 10A-like |
|  | Pbr027730.1 | Up | Protein TIFY 10A |
|  | Pbr028362.1 | Up | Protein TIFY 6B |
|  | Pbr018411.1 | Up | Transcription factor MYC2 like |
|  | Pbr042466.1 | Up | Transcription factor MYC2 |
|  | Pbr009479.1 | Up | Coronatine-insensitive protein 1-like |
|  | Pbr021060.1 | Down | Jasmonoyl-L-amino acid synthetase JAR4 isoform X3 |
| Auxin | Pbr012348.1 | Down | Auxin transporter-like protein 2 isoform X1 |
|  | Pbr028070.1 | Up | Auxin response factor 5-like |
|  | Pbr025290.1 | Down | Auxin-induced protein AUX28-like |
|  | Pbr025212.1 | Up | Probable indole-3-acetic acid-amido synthetase |
|  | Pbr033623.1 | Up | Auxin-responsive protein SAUR71-like |
| Abscisic acid | Pbr030476.1 | Up | G-box-binding factor 4-like |
|  | Pbr028222.1 | Up | Abscisic acid receptor PYL4-like |
|  | Pbr006341.1 | Down | Serine/threonine-protein kinase SAPK2-like |
|  | Pbr009416.1 | Up | Protein phosphatase 2C 37-like |
|  | Pbr027457.1 | Down | Abscisic acid receptor PYL9 |
| Cytokinin | Pbr035647.1 | Up | Histidine kinase 3-like |
|  | Pbr011180.1 | Down | Histidine-containing phosphotransfer protein 1-like |
|  | Pbr020017.1 | Up | Two-component response regulator ARR12 isoform X2 |
|  | Pbr039183.2 | Down | Two-component response regulator ARR6 |
|  | Pbr015157.1 | Up | Histidine kinase 3 isoform X1 |
| Ethylene | Pbr023044.1 | Up | Ethylene-responsive transcription factor 1B-like |
|  | Pbr024739.1 | Up | ETHYLENE INSENSITIVE 3-like 1 protein |
|  | Pbr027843.1 | Up | EIN3-binding F-box protein 1-like |
|  | Pbr002199.1 | Up | Ethylene receptor 2-like precursor |
|  | Pbr010645.1 | Up | EIN3-binding F-box protein 1-like |
| Brassinosteroid | Pbr004288.1 | UP | BRASSINOSTEROID INSENSITIVE 1-associated receptor kinase 1-like |
|  | Pbr021932.1 | UP | Brassinosteroid LRR receptor kinase-like |
|  | Pbr000513.1 | UP | Shaggy-related protein kinase |
|  | Pbr025537.1 | Down | Cyclin-D3-1 |
|  | Pbr000888.2 | UP | BRASSINOSTEROID INSENSITIVE 1-associated receptor kinase 1-like |
