# Supplementary material for: Transcriptome analysis provides new insights into the transcriptional regulation of methyl jasmonate-induced flavonoid biosynthesis in pear calli
Source: BMC Plant Biol. 2020 Aug 25;20:388. doi: 10.1186/s12870-020-02606-x (PMC7446162; doi:10.1186/s12870-020-02606-x)
Supplement: Supplementary file 7 — Additional file 7: Table S4. Primers used for the qRT-PCR analysis. [file 12870_2020_2606_MOESM7_ESM.docx]

| Gene | | Forward primer (5'to 3') | Reverse primer (5'to 3') |
| --- | --- | --- | --- |
| Pbr020913.1 *(PcCHS*) | CAAGCGCCTCATGATGTACC | | CAAGGCTTGACCCACAAGAC |
| Pbr034840.1 (*PcF3H*) | AGGGGACGAAGAAGTACAGC | | CAAGAGTTAGGTCGGGCTGA |
| Pbr005931.1(*PcDFR*) | CACTGGACTTTCGCCGATCT | | ACAAATGTAGCGACCCTCGG |
| Pbr001543.1 (*PcANS*) | GGGAGGCTGGAGAAAGAAGT | | CTTGGCAGTGACCCACTTTC |
| Pbr013248.1 (*PcLAR1*) | ACCCTTCTAAGGCTGACACC | | TGGCGTCGACTAGGGTAATC |
| Pbr016663.1 (*PcMYB10*) | CAGCAGAAGATTTAAGTACGCCATC | | TTCTAACAAGGTCTCCCACCAATC |
| Pbr015228.1(*PcMYB79*) | GGACCGCAGAAGAAGATCAG | | CCGGCAATCAAAGACCACC |
| Pbr031682.1(*PcMYB13*) | CTGGGGAACAGATGGTCTCT | | CAAGGGATGATGGTGATGCC |
| Pbr034465.1(*PcMYB17*) | TTGGAAGCAGGTGGTCTGTC | | TGTTGTTTACGCTGCTTGCC |
| Pbr009479.1 (*PcCOI1*) | GAGGACTGGGGAGGGTATGT | | AACCCTGAGCACTTGTCGAG |
| Pbr021060.1 (*PcJAR1*) | CGTGGAGTACTTGCAGGGTT | | CCAGTGAGGATGGGAGAGGA |
| Pbr027730.1(*PcJAZ1*) | CCTAGCACCGTACCAAACGA | | CATTGCAGAGGCTTGAACCG |

**Additional file 7: Table S4: Primers used for qRT-PCR analysis**
