# Supplementary material for: Transcriptome analysis provides new insights into the transcriptional regulation of methyl jasmonate-induced flavonoid biosynthesis in pear calli
Source: BMC Plant Biol. 2020 Aug 25;20:388. doi: 10.1186/s12870-020-02606-x (PMC7446162; doi:10.1186/s12870-020-02606-x)
Supplement: Supplementary file 8 — Additional file 8: Table S5. Accession numbers of the flavonoid regulatory MYB transcription factors in pear (Pyrus spp.), apple (Malus domestica), and Arabidopsis (Arabidopsis thaliana) that were included in the phylogenetic analysis. [file 12870_2020_2606_MOESM8_ESM.docx]

| Species | Gene | Accession |
| --- | --- | --- |
| Pear (*Pyrus* spp.) | PpMYB114  PbMYB12b  PbMYB9  PbMYB10b | MF489219  LOC103931986  KT601123  KT601122 |
| Arabidopsis | AtMYB4 | AT4G38620.1 |
| (*Arabidopsis thialana*) | AtMYB11 | AT3G62610.1 |
|  | AtMYB12 | AT2G47460.1 |
|  | AtMYB32 | AT4G34990.1 |
|  | AtMYB46 | AT5G12870.1 |
|  | AtMYB60 | AT1G08810.1 |
|  | AtMYB61 | AT1G09540.1 |
|  | AtMYB75/PAP1 | AT1G56650.1 |
|  | AtMYB90/PAP2 | AT1G66390.1 |
|  | AtMYB111 | AT5G49330.1 |
|  | AtMYB113 | AT1G66370.1 |
|  | AtMYB114  AtMYB123/TT2  AtMYBL2 | AT1G66380.1  AT5G35550.1  NM_105772 |
| Apple | MdMYB1 | ADQ27443.1 |
| (*Malus domestica*) | MdMYB3 | AEX08668.1 |
|  | MdMYB6 | AAZ20429.1 |
|  | MdMYB10 | AFC88038.1 |
|  | MdMYBA | BAF80582.1 |
|  | MdMYB22 | AAZ20438.1 |
|  | MdMYB12 | XP_008337875.1 |

**Additional file 8: Table S5: Accessions of flavonoid regulatory MYB transcription factors in Pear (*Pyrus* spp.), Arabidopsis (*Arabidopsis thialana*) and apple (*Malus domestica*) used for Phylogenetic analysis**
